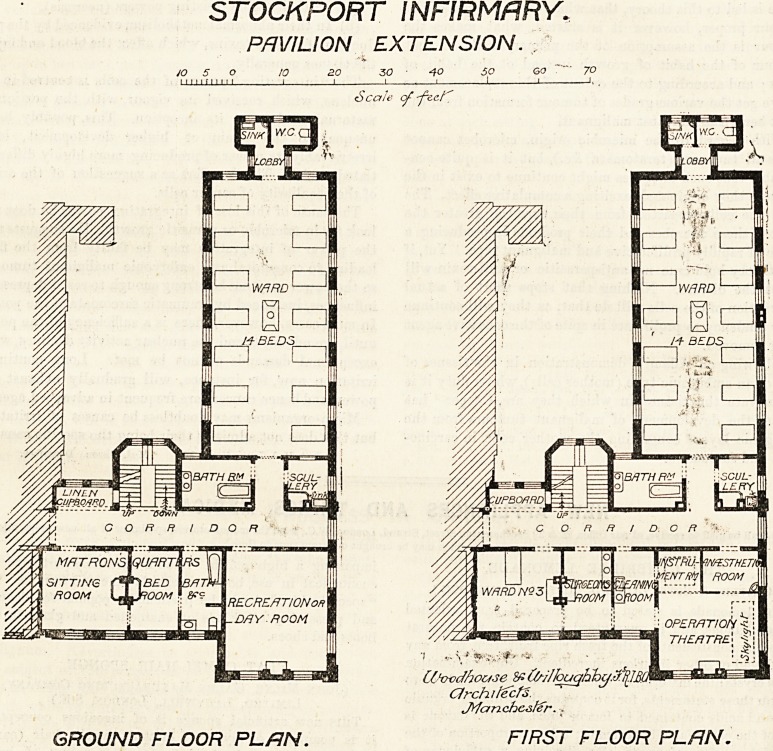# Stockport Infirmary Additions

**Published:** 1901-05-25

**Authors:** 


### The Institutional Workshop.

### STOCKPORT INFIRMARY ADDITIONS.

A large pavilion block has been built at this infirmary as a memorial of the Diamond Jubilee, and it was formally opened a few months since by Lord Newton.

The new pavilion consists of four stories, and it is built in line with the older part of the infirmary, thus completing the façade towards Wellington Road. The block covers an area of about 420 square yards, and in addition to this block, which contains all the new wards and the operating theatre, there have been provided a laundry, wash-house, post-mortem room, and mort uary. Thelatter cover an area of 230 square yards, and are placed in a block at the rear of the main building and at right angles to it. The walls of the post-mortem room are entirely lined with white glazed bricks, and a specially designed table of slabs has been put up. Over this table is a skylight, which will serve the double purpose of giving sufficient light and of ensuring easy ventilation.

Returning to the pavilion it will be seen that a direct means of access to every part has been obtained by a well-lighted corridor and stone staircases opening on to each floor of the block.

On the *lower* ground floor, and immediately under the wards, is a recreation room for the patients. The size of this room is 54 feet by 23 feet. Considering the undoubted usefulness of these recreation rooms in our hospitals, it is a matter of wonder and regret that such adjuncts are not more generally provided. This lower ground floor also contains bathrooms for the resident medical staff, storerooms, and a heating chamber, the latter being approached from the outside.

On the *upper* ground floor there is a ward 54 feet long and 23 feet wide. This ward is well lighted and ventilated, and has attached to it nurses' scullery, lavatory, bathroom, and closets, the latter being cut off by cross-ventilated passages. The ward contains 14 beds. On this floor we also find a patients' day-room and quarters for the resident surgeon.

On the first floor is another ward for 14 patients, with offices of similar description to those on the ground floor. There is also on this floor a small ward for special cases, and this room is close to the operating theatre. The latter is placed on the opposite side of the corridor and is contiguous to the main staircase. The theatre is most properly provided with both side-light and top-light, and it has, as adjuncts, anæsthetic room, instrument-room, and surgeons' room, all *en suite*. These rooms have ceramic mosaic floors, and the walls are lined with glazed tiles. The supply pipes to all the sinks are of copper: they are fixed on the surfaces of the walls, and not embedded as they often, very foolishly, are, and the waste pipes are carried off in the best known manner. These fittings have been specially designed, for their respective purposes, from particulars supplied by the medical committee of the infirmary. The theatre is warmed and ventilated on Blackman's system.

The second floor of the pavilion contains nurses' bedrooms, bath-rooms, and lavatories.

The new building is throughout of fire-proof construction. The walls are finished in Keen's cement, and the angles of the walls have been done away with; an omission which avoids the accumulation of much dust and simplifies the cleaning process.

We are glad to notice that open fireplaces are provided, although they are supplemented by Beeley's hot-water system of warming. The floors of the wards are of polished oak, and the boards are narrow and are secret-nailed. The windows are all fitted with patent sashes, made so that they can be drawn inwards and be easily and safely cleaned.

The architects are Messrs. Woodhouse & Willoughby, of Manchester. The general contractors were Messrs. Meadows, of Stockport, but subsidiary contracts were held by Messrs. Twyford, of Hanley, for fittings; by Messrs. Doulton, for grates; by Messrs. Fletcher & Russell, of Pendleton, for gas ranges; by Messrs. Roger Lowe, of Farnworth, for flooring; by Messrs. Williams, of Manchester, for mosaic floors; and by Messrs. Marley Bros., of Birmingham, for ironmongery. The total cost was £12,000.

## Figures and Tables

**Figure f1:**